# Pressure and Temperature Changes in *In Vitro* Applications with the Laser and Their Implications for Middle Ear Surgery

**DOI:** 10.1155/2010/237521

**Published:** 2010-10-04

**Authors:** Burkard Schwab, Georgios Kontorinis

**Affiliations:** Department of Otolaryngology, Medical University of Hannover, Carl-Neuberg-Straße 1, 30625 Hannover, Germany

## Abstract

*Background*. The purpose of this study was to evaluate the thermal and pressure effects using a Titan Sapphire chirped-pulse amplifier system configured to deliver ultrashort pulses of 180 femtoseconds (fs) in an inner ear model. *Materials and Methods*. Temperature increases and heat exchange processes in the fluid (physiological saline) were examined in a calorically and physiologically approximated cochlea model for applying laser parameters effective in the creation of footplate perforations. *Results*. In the effective energy density range, the highest temperature increases achieved with the Carbon dioxide (CO_2_) laser were about 11 degrees C. The lowest temperature maxima were 6 degrees C with the Er:YAG laser (Yttrium-Aluminum-Oxide doted with Erbium3+-ions) and <5 degrees C with the femtosecond laser. Comparison of the laser-induced pressure with the limit graph published by Pfander indicated that the use of the fs laser is unobjectionable for fluences <1 J/cm^2^. *Conclusions*. Our investigations demonstrated that the application of the fs laser in middle ear surgery presents a new and promising addition to the range of ultrashort wavelength lasers used for this purpose.

## 1. Introduction

Since the first stapedectomies were carried out by Shea in the late 1950s [1], otosclerosis surgery has become a routine procedure. Following the initially purely mechanical treatment of the stapes suprastructure and footplate, various laser systems have seen increasing use since the 1980s, aiming at development of contactless techniques. As early as 1967, Sataloff [2] reported on the experimental application of laser (a neodymium glass laser) for an *in vitro* stapedectomy. Perkins [3] was the first to perform a stapedectomy using the argon laser, although the footplate of the stapes was only partially ablated; after having been reduced in thickness by the laser, the footplate underwent further mechanical fracturing. In the following years there were reports of numerous successful operations with good audiological results, for example, from Di Bartolomeo and Ellis [4], McGee [5], Silverstein et al. [6], and Lesinski and Palmer [7]. 

Thermal laser systems such as argon, carbon dioxide (CO_2_), and KTP (potassium titanium oxide phosphate) laser have been dominant in stapes surgery up to now; however, it is precisely the thermal stress and the associated potential for damage to the inner ear structures [8–10] or the facial nerve [11, 12] that are a property of these systems. Even in pulsed lasers such as the Er:Yag laser (Yttrium-Aluminum-Oxide doted with Erbium3+-ions), which involve a subtotal absorption of laser light by bone, the explosive nature of the ablative process incurs the risk of acoustic damage [13, 14].

**Figure 1:**
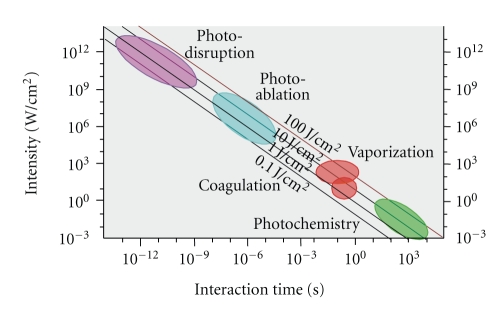
Schematic representation of laser-tissue interactions in relation to exposure time and input energy.

The femtosecond laser with its ultrashort pulse (1 fs = 10^−15^ s) may be able to minimize or avoid these effects. In tissue that is exposed to ultrashort pulses so-called multiphoton processes occur (i.e. the simultaneous absorption of several photons) which bring about optical rupture and plasma formation. This plasma-induced photodisruption (Figure 1) enables cell material to be ablated with great precision. The extremely short pulses lead to a minimization of the collateral damage, both thermal and mechanical, to the surrounding tissue [3].

Femtosecond lasers are finding many uses in the medical field ranging from biopsy imaging to eye surgery. One of the first commercially successful applications of femtosecond lasers is their use in the LASIK (Laser in situ keratomileusis) eye surgery procedure. The ultrafast laser replaces the microkeratome mechanical knife that makes the initial cut in the cornea. This offers a highly controlled cut of uniform thickness, which is not possible with the mechanical knife [15]. Work has also been done using femtosecond lasers to treat atherosclerosis. The buildup of plaque causes arteries to harden, restricting blood flow. By ablating tissue from the artery wall the elasticity of the artery can be restored. Blood pressure forces the artery to expand once wall material has been removed. This procedure could replace in future balloon angioplasty or stenting procedures, as the use of laser ablation offers the advantage of being less damaging to the structural integrity of the artery than other procedures. Furthermore, dental surgeries are areas where the extremely clean material processing abilities of femtosecond lasers offer an alternative to mechanical drills or CW lasers that leave microcracks and cause thermal stress in tooth enamel [15].

The outstanding precision of femtosecond laser could be demonstrated by analyzing laser generated cavities in human ossicles by electron microscopy. Even at pulse repetition rates of 3 kHz, no thermal effects like melting zones could be found. At the cavities wall, still the opened bone channels could be recognized [15].

Aim of the present study was to assess how far these expectations are fulfilled for a cochlear model, with special reference to any thermal damage.

## 2. Material and Methods

The investigations were carried out using a Titan-sapphire laser. Based on the principle of passive “Kerr lens mode locking” [16] this laser generates a femtosecond burst of light (100 Mhz) with a pulse width of <100 fs. The beam is then defined (or “chirped”) in a pulse stretcher which extends the frequency cycle to 200 ps. Transmission to the amplifier and the compressor connected to it then generates energy of between 1 *μ*J and 1 mJ, with a pulse frequency of <200 fs at a central wavelength of 780 nm and a repetition rate of max. 1.04 kHz.

For modeling purposes the cochlea was regarded as a fluid-filled target space (endolymph) embedded in a solid body with decelerated heat transfer (bone). The thermal adjustment of this open system when subjected to laser bombardment depends on laser-induced heat input and heat conduction to the environment; convection and radiation are relatively insignificant.

**Figure 2:**
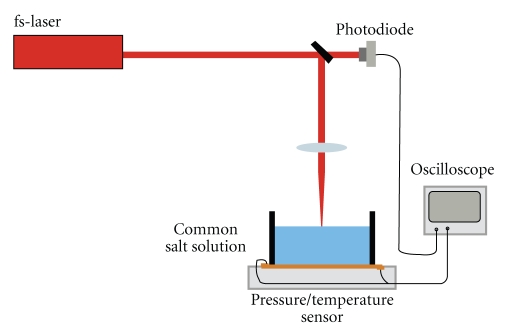
Plexiglas model of the inner ear with a target volume of 0.3 ml

The present system was based on that used by Jovanovic in studies involving inner ear model [9, 17]. Temperature changes in the cochlea were simulated using a drilled polymethylmethacrylate (PMMA) body filled with 0.3 ml of water. The rise in temperature of the target space upon laser bombardment was registered with the aid of a temperature sensor (compensated NTC) (Figure 2). 

The laser parameters used are given in Table 1. The model was then subjected to further experiments to determine the compressive load on the inner ear. The fs laser was operated at a repetition rate of 10 Hz. The pressure sensor, made of polyvinylidene fluoride (PVDF) film and with a sensitive surface area of 1 mm², has a response time of 5 ns.

The laser beam was focussed on the fluid level of the inner ear model. In addition to the femtosecond, other lasers were used, namely, the Er:YAG laser (OPMI Twin Er, wavelength: 2.94 *μ*m, focus: 1/*e* ^²^:380 *μ*m, Carl Zeiss, Oberkochen, Germany) and the CO_2_ laser, delivered by micromanipulator (Sharplan 20C SilkLaser, 10.6 micron, Lumenis, Inc., Santa Clara, California, USA), which is known to generate the highest temperature [18]. Due to the numerous reports on the KTP laser and the already existing comparisons of its thermal effect with the above mentioned types in vitro [6, 7, 12], KTP laser was not used in our study. 

The above-mentioned experimental conditions simulated the situation following perforation of the footplate, that is, direct energy input into the perilymph. The laser parameters used had already been determined in preliminary experiments [19] to perform a complete perforation of the footplate.

## 3. Results

The increase of the temperature recorded over the duration of  laser exposure was influenced by the mixing process within the measuring chamber and the emission of heat to the surroundings. The temperature reached its peak when the input energy flow and the outward heat flux were equal.

**Figure 3:**
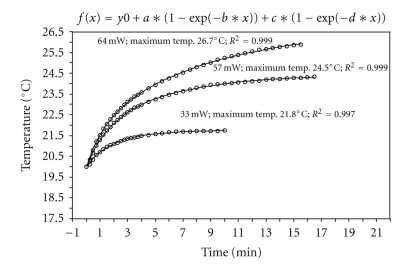
Temperature change in H_2_O with the Er:YAG laser (33 mW at 3.7 Hz; 57 mW at 3.7 Hz, 63 mW at 3 Hz).

**Figure 4:**
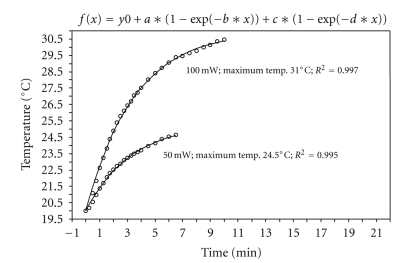
Temperature change in H_2_O with the CO_2_ laser, pulsed, repetition rate 4 Hz.

**Figure 5:**
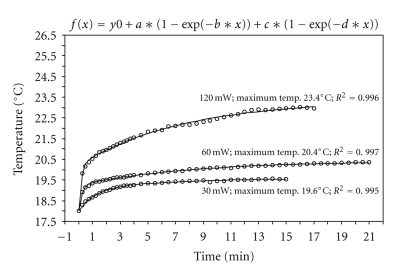
Temperature change in H_2_O with the fs laser (100 fs, 3 Hz).

By means of double exponential adaptation, a mathematical model was devised that allowed temperature change to be accurately predicted. The peak value so determined (labeled “maximum temperature” in the curves shown in Figures 3, 4, and 5) allowed a direct comparison between the heat input for the various lasers and settings.

The CO_2_ laser generated the highest temperature increases over time. Even with energy input of only 50 mW the temperature rose after some 7 minutes to a maximum of 5.5°C above the initial value. Doubling the energy increases the maximum temperature reached after 10 minutes by approximately 11°C (Figure 4).

Lower temperature increases were recorded with the Er:YAG laser: at an energy input of 33 mW and a repetition frequency of 3.7 Hz the absolute temperature increase was 1.8°C, rising to 4.5°C at 57 mW and, at the maximum energy level of 64 mW, to 6.7°C (Figure 3). 

The lowest temperature increase we recorded was for the fs laser: at an energy input of 30 mW the temperature increases by 1.6°C, at energies of 60 mW the resultant increase in peak temperature is 2.4°C; even at the highest energy input level of 120 mW the temperature increased by only 5.4°C (Figure 5).

**Figure 6:**
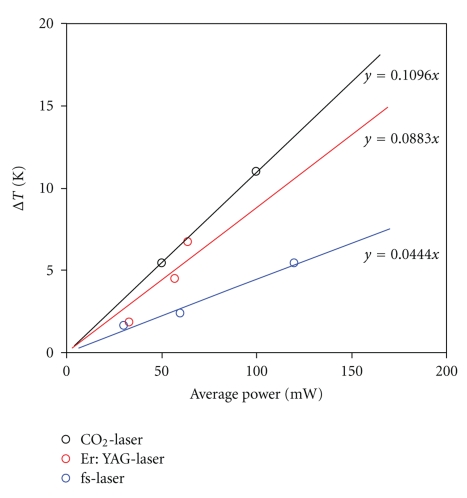
Temperature increases using different laser systems.

Figure 6 shows the extrapolated steady-state temperature increase as a function of input laser power for the laser types investigated. The gradient of the “averaging” line drawn between the points yields the heat input ratio for the various laser types (Figure 6).

The pressure load on the inner ear was determined in further model-based investigations.

The fs laser was operated at a repetition rate of 10 Hz. The pressure sensor, made of PVDF film and with a sensitive surface area of 1 mm², has a response time of 5 ns.

**Figure 7:**
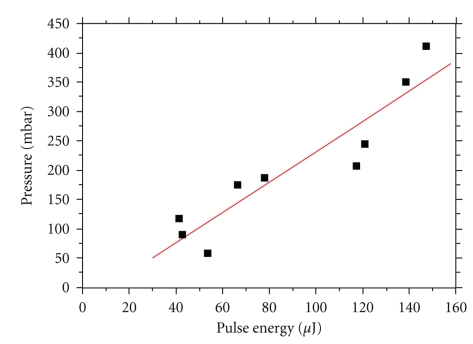
Linear increase in pressure in relation to pulse energy.

Figure 7 shows the linear increase of pressure in relation to input energy; pressure impulse values of 600 mbar, reported by Pfander [20] as potentially causing inner ear damage, were not reached.

**Figure 8:**
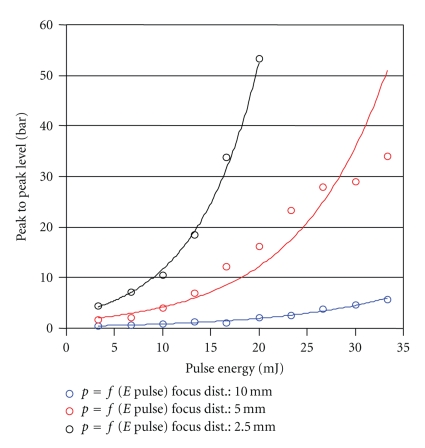
Peak pressure in relation to pulse energy using the Er:YAG laser, at varying focal distance from the measuring element.

**Figure 9:**
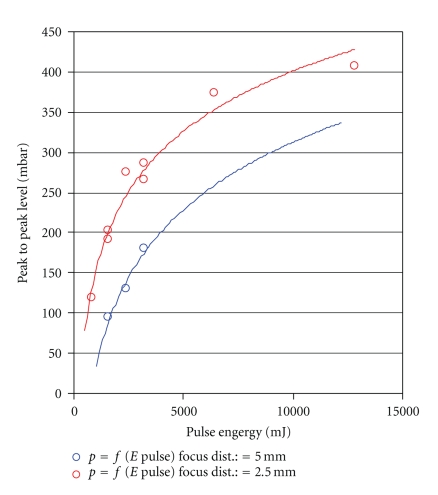
Peak pressure in relation to pulse energy using the CO_2_-Laser at varying focal distance from the measuring element.

**Figure 10:**
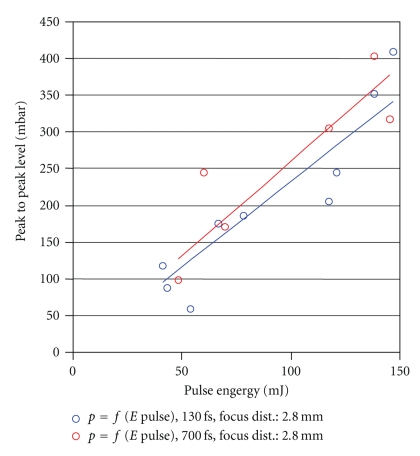
Peak pressure in relation to pulse energy using the fs laser, at varying fs pulse length.

Comparison of the recorded peak pressures with those of the Er:YAG laser, currently used in middle ear surgery, shows substantial reduction of the pressure values, with much lower energy input, as well (Figures 8, 9 and 10). Both, the energy values and the pressure pulse associated with the fs laser are an order of magnitude lower.

## 4. Discussion

Since pioneering work on stapes surgery began at the end of the 19th century, one of its chief aims has been to minimize the inner ear trauma. When laser stapedotomy was first performed by Perkins [3] in the late 1970s, there were high hopes for a further reduction in the side effects of a purely mechanical manipulation of the footplate or stapes suprastructure.

Since that time, the lasers used have mainly been thermal in nature, such as the Argon, KTP, or CO_2_ laser. Whereas in the emerging years of laser stapedotomy, the response to the argon laser verged on the euphoric [3, 4]; use of this very system (together with the KTP laser) led to not inconsiderable temperature increases of the perilymph upon direct irradiation of the open vestibulum. For this reason the two laser types were rated as unsuitable for stapes surgery by Lesinski and Palmer [7] and Jovanovic et al. [21]. The argon laser was also associated with a threefold increase in the occurrence of postoperative giddiness [6].

Subsequently, the Excimer laser, with a wavelength of 193 nm, was also introduced [22], followed by the Er:YAG and CO_2_ lasers [14]. Direct comparison shows all laser types to have specific advantages and disadvantages: whereas temperature increases are, accordingly, greater for the CO_2_ laser [18], higher peak pressures can be expected with the Erbium laser [8, 14, 23]. The two latter systems have nevertheless become well established in routine clinical practice and have seen wide distribution and acceptance.

The lower energy inputs now possible with the fs laser mean that both problems could be circumvented. Not only are the maximum temperature increases associated with *in vitro* bombardment of the perilymph substantially lower than those for conventional laser systems (especially the Er:YAG, which itself tends to be seen as a “cold” laser [8, 14, 23]), but the peak pressure is also well below the levels generated by the commonly used Er:YAG laser.

In this respect, the concept of femtosecond laser application combines the advantages of the various laser types while dispensing with their respective disadvantages. Laser types currently used in middle ear surgery are either based on the principle of continuous wave technology or use relatively long pulse durations. In the latter case, however, the ablation process is dependent on the thermal and optical properties (such as thermal diffusion and absorption coefficient) of the material. The two parameters of wavelength and pulse duration therefore represent limiting factors. Owing to the low absorption of laser energy in water or bone within the visible green range [24], the KTP (Nd:YAG) laser (*λ* = 532 nm), or the Argon laser (*λ* = 514 nm) appear to be suitable for middle ear and stapes surgery, respectively, as the expected generation of heat does not occur [25]. There is, however, the risk that deeper-lying structures will be damaged owing to poor absorption in water (or perilymph). Lasers with infrared wavelengths, such as the CO_2_ laser (*λ* = 10.6 *μ*m) or the Er:YAG laser (*λ* = 2.94 nm), achieve a high absorption rate in bone and surrounding tissue [7, 26] although this may be accompanied by the generation of relatively large amounts of heat [27]. 

In contrast, when ultrashort pulses are applied, the ablation process is virtually independent of both, the material properties and the wavelength used. Moreover, the mechanical and thermal side effects are far less severe, since a large portion of the input energy is carried away with the ablated tissue [7]. The total input energy used for ablation is considerably lower than with conventional laser systems. 

Indeed, whereas the Er:YAG laser requires total energy of 0.35–0.75 J for footplate perforation and the CO_2_ laser of 0.2–1.8 J (depending on pulse duration) [9, 27], the energy values when ultrashort pulses are applied are of the order of millijoules (mJ). Although CO_2_ laser was reported to be safe when used in revision stapes surgery with fluoroplastic wire pistons, application of 6 W to stainless steel pistons can disturb the inner ear function [28]. Furthermore, previous work described delayed facial nerve palsy as a result of heating after KTP laser stapedectomy [29]. In the same study, further *in vitro* investigation revealed maximum temperature values in the facial canal between 1.4°C and 15.2°C, values much higher than those recorded with fs laser in our experimental setting. 

Based on the above values, it can be inferred that for a given radiated power output, the fs laser releases only half the amount of heat emitted by the Er:YAG laser and less than CO_2_ and KTP laser; it appears that thermal damage of the surrounding tissue can be virtually ruled out or at least minimized. This leads in turn to a substantial reduction in undesirable tissue reactions. The technique of multiphoton ablation allows precise spatial definition of the tissue ablation process, as it is entirely restricted to the focus area. The risk that direct laser bombardment will cause damage to deeper-lying structures (such as the utricle and saccule) following footplate perforation is thereby significantly reduced. 

A drawback of the fs laser that has received criticism in this connection is that it is not conducive to coagulation. The extremely low temperature increase during the multiphoton ablation process means that there is no coagulation effect acting on the (possibly bleeding) mucosa. This disadvantage can, however, be offset by the use of vasoconstrictive substances applied to the footplate or to the ossicles, whenever required.

## 5. Conclusions

Our investigations demonstrated that the application of the fs laser in middle ear surgery presents a new and promising addition to the range of ultrashort wavelength lasers used for this purpose.

The concept of femtosecond laser application combines the advantages of the various laser types while dispensing with their respective disadvantages. This means that owing to the much lower energies required close to the threshold when tissue is ablated using the fs laser, the resulting pressure load on the inner ear—and therefore the potential damage—can be greatly reduced.

Further studies are currently in preparation; predominantly using animal models. These are aimed at reviewing the effectiveness and safety of fs application. With the advent of this new technology in routine clinical practice, it can be expected that the system will undergo miniaturization, since clinical use is still prevented by the size of the laser setup.

## Figures and Tables

**Table 1:** Laser parameters used for the various laser types.

Laser type / *λ*[nm]	Focus diameter[*μ*m]	Mean output power[mW]	Pulse energy[mJ]	Pulse duration	Repetition rate[Hz]
CO_2_ / 10640	300	50	12,5	50 ms	4
		100	25	50 ms	4
Er:YAG / 2940	200	33	9	85 *μ*s	3,7
		57	15	110 *μ*s	3,7
		64	21	130 *μ*s	3
fs laser / 780(100 fs)	80	30	10*10^−3^	100 fs	3000
		60	20*10^−3^	100 fs	3000
		120	40*10^−3^	100 fs	3000
